# Measuring biological aging in humans: A quest

**DOI:** 10.1111/acel.13080

**Published:** 2019-12-12

**Authors:** Luigi Ferrucci, Marta Gonzalez‐Freire, Elisa Fabbri, Eleanor Simonsick, Toshiko Tanaka, Zenobia Moore, Shabnam Salimi, Felipe Sierra, Rafael de Cabo

**Affiliations:** ^1^ Translational Gerontology Branch Biomedical Research Center National Institute on Aging National Institutes of Health Baltimore MD USA; ^2^ Department of Medical and Surgical Sciences University of Bologna Bologna Italy; ^3^ Department of Epidemiology and Public Health University of Maryland School of Medicine Baltimore MD USA; ^4^ Division of Aging Biology National Institute on Aging NIH Bethesda MD USA

**Keywords:** aging, biological aging, hallmarks of aging, inflammation, multimorbidity, resilience, senescence

## Abstract

The global population of individuals over the age of 65 is growing at an unprecedented rate and is expected to reach 1.6 billion by 2050. Most older individuals are affected by multiple chronic diseases, leading to complex drug treatments and increased risk of physical and cognitive disability. Improving or preserving the health and quality of life of these individuals is challenging due to a lack of well‐established clinical guidelines. Physicians are often forced to engage in cycles of “trial and error” that are centered on palliative treatment of symptoms rather than the root cause, often resulting in dubious outcomes. Recently, geroscience challenged this view, proposing that the underlying biological mechanisms of aging are central to the global increase in susceptibility to disease and disability that occurs with aging. In fact, strong correlations have recently been revealed between health dimensions and phenotypes that are typical of aging, especially with autophagy, mitochondrial function, cellular senescence, and DNA methylation. Current research focuses on measuring the pace of aging to identify individuals who are “aging faster” to test and develop interventions that could prevent or delay the progression of multimorbidity and disability with aging. Understanding how the underlying biological mechanisms of aging connect to and impact longitudinal changes in health trajectories offers a unique opportunity to identify resilience mechanisms, their dynamic changes, and their impact on stress responses. Harnessing how to evoke and control resilience mechanisms in individuals with successful aging could lead to writing a new chapter in human medicine.

## Graphical abstract

Finding a reference metric for the rate of biological aging is key to understanding the molecular nature of the aging process. Defining and validating this metric in humans opens the door to a new kind of medicine that will overcome the limitation of current disease definitions. We will then be able to approach health in a global perspective and bring life course preventative measures to the center of attention.

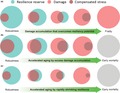

## 1 INTRODUCTION

**Figure 1:**
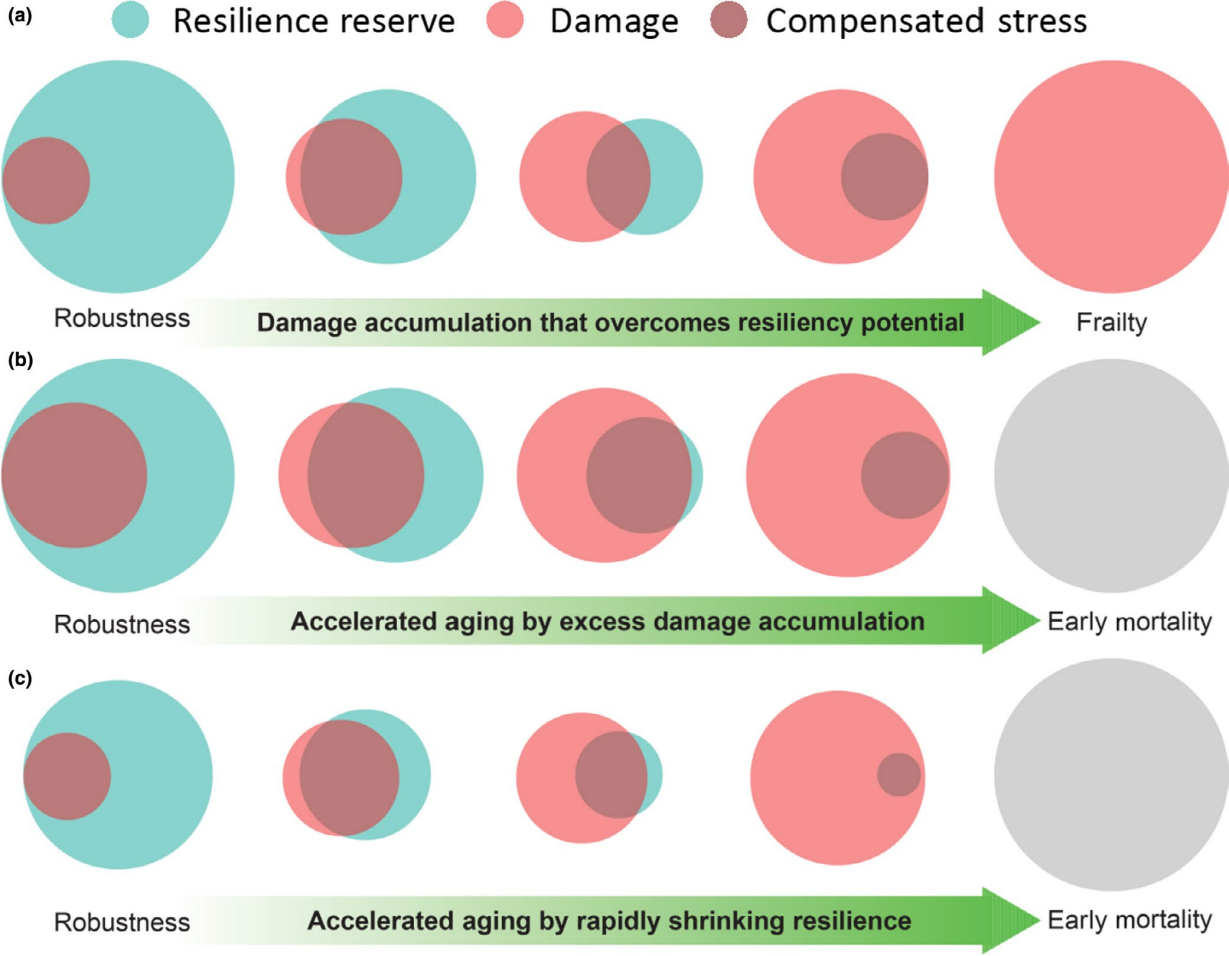
Normal aging (a) and different pathways to accelerated aging (b and c). A. Robust resilience at a young age fully compensates damage. Over time, damage accumulates that is not fully compensated by resilience. Toward the end of life, resiliency is overwhelmed, and new stresses cause fast, unopposed damage accumulation that leads to frailty and eventually to death. Accelerated aging may occur either because of faster rates of damage accumulation (b) or because of rapid shrinking and eventual collapse of resilience (c). Note that even in the state of robustness, damage can be already abnormally high (b) and resilience already abnormally low (c)

In its most profound essence, resilience is at the core of life and is interpreted as the harmonic assemblage of the biochemical processes that are aimed at maintaining the identity, integrity, and autonomy of individual organisms against the perturbations induced by both internal and external environments. Developmental changes that occur during fetal growth and postnatal development are fast, massive, tightly predetermined, and stereotyped, probably because they are driven by a redundant and self‐correcting genetic “software.” This period of development is followed by a time of relative stability, where changes in physical and cognitive function are small and only detectable by very sensitive tools or challenging tests. During this “middle” period, most individuals in the population are free of diseases (Blekhman et al., 2008; Olshansky, 2016). However, underneath this apparent stability there are several compensatory and homeostatic mechanisms hidden that constantly operate to preserve biochemical balance and prevent phenotypic derangements, as well as functional decline. Early in life, these mechanisms are highly effective and provide a robust homeostasis, but begin to fade later in life, and unrepaired damage accumulates beyond the functional threshold (Figure 1). The extreme variability by which these mechanisms maintain a stable homeostasis explains why the variance of aging phenotypes expands over time, even at extreme old age despite the leveling force of selective mortality. Understanding the nature of these “resilience mechanisms” (homeostatic mechanisms, in green in Figure 1) and “accumulated damages” (entropic forces, in red in Figure 1), as well as finding methods to assess them in humans, is a very active area of investigation. For example, while the condition of “frailty” in older persons is often defined as a “reduction of physiological compensation,” almost all criteria currently proposed are based on measures of damage. Damage only emerges clinically when compensatory mechanisms are exhausted (Ferrucci & Fabbri, 2018). As shown in Figure 1, physical decline, cognitive decline, and frailty may result from two interrelated mechanisms, one inducing and the other preventing damage, which may act separately or jointly. We postulate that the interaction between damage and repair could explain why some individuals are aging “faster” and studying them jointly may point to the mechanisms of accelerated aging.

Studies in animal models have begun to reveal the nature of these mechanisms, and some assays for humans have been developed. Although many of these unique or composite measures generally track chronological age with a predictable schedule, the biology of their compensatory and homeostatic nature is only partially explained and their relevance for health is limited to observational studies (Hilmer & Le Couteur, 2016; Kirkland, Tchkonia, Zhu, Niedernhofer, & Robbins, 2017; Moreno‐Villanueva et al., 2015; Newman et al., 2016; Niedernhofer, Kirkland, & Ladiges, 2017; Richardson et al., 2015; Robbins & Niedernhofer, 2017).

In the next part of this manuscript, we will try to summarize what measures of aging biology are currently available. We are inspired by two recent articles that outlined the “hallmarks” and the “pillars” of aging (López‐Otín, Blasco, Partridge, Serrano, & Kroemer, 2013; Sierra, 2016), but we purposely limit this description to those measures that can be obtained in humans and we point to their validity, limitations, and potential for further development. For most biomarkers, whether they reflect damage, compensation, or a combination of the two remains unknown.

### 1.1 Genomic instability

The accumulation of DNA damage (somatic mutations) with age has been proposed as the primary cause of aging because of its effects on the fidelity of proteins and the regulation of gene expression. While mutational load plays a role in carcinogenesis, solid evidence that the accumulation of somatic mutations during normal aging is associated with the phenotypes of aging is lacking. Studies that compare single‐cell and multicellular DNA high‐fidelity sequences and studies that systematically screen for mutation in single cells that are clonally expanded are underway. Spontaneous somatic mutations accumulate in human B lymphocytes, and it has been suggested that they may contribute to functional decline of B lymphocytes in the elderly (Zhang et al., 2019). Similarly, a slight accumulation of DNA somatic mutations with aging has been demonstrated in skeletal muscle satellite cells from human biopsies (Franco et al., 2018), while Bae et al. sequenced DNA from single neurons and demonstrated that somatic mutations accumulate with aging from 4 months to 82 years of age (Bae et al., 2017; Lodato et al., 2017). The functional relevance of these mutations is unknown.

While several biochemical and cell‐based tests of DNA repair capacity have been developed and shown to be reasonably objective and reliable, quantification of DNA repair capacity in humans remains unsatisfactory (Berwick & Vineis, 2005; Trzeciak et al., 2008; Trzeciak, Barnes, & Evans, 2008). The few tests described in the literature have not been applied to large populations and lack independent validation (El‐Zein et al., 2010; Fang, Neutzner, Turtschi, Flammer, & Mozaffarieh, 2015; Hamann & Hartwig, 2014; Holton, Ebenstein, & Gassman, 2018; Nagel et al., 2014; Reddy et al., 2016). Moreover, there is no consensus on gold standard assays and most methods require large amounts of freshly collected pure cell types, and these only address repair capacity of a subset of specific lesions. For example, the comet assay, which quantifies alkaline‐labile sites and/or specific DNA strand breaks, has been used for years, but the reliability and validity of its results have been questioned, partly due to extreme sensitivity to experimental conditions (Collins, 2014; Saha et al., 2008). In addition, some assays require repair of an exogenous substrate, but the substrate design has been proven challenging (Latimer & Kelly, 2014; Reddy et al., 2016; Shen, Fox, Ahn, & Loeb, 2014). DNA somatic mutations accumulation and loss of efficiency of DNA repair mechanisms are likely important drivers of biological aging. However, reliable and valid assays for their quantification should be developed before they can be used in human research and in clinical applications.

### 1.2 Telomere length

Telomeres are tract of tandem repeats of the six‐nucleotide unit sequence (TTAGGG) that protect chromosome ends from eliciting a DNA damage response. During DNA replication, the DNA polymerases are unable to fully recreate the end of the telomeric DNA and telomeres shorten during each cell division, which ultimately leads to replicative senescence in vitro (Allsopp et al., 1992; Greider, 1998; Herbig, Jobling, Chen, Chen, & Sedivy, 2004). The enzyme telomerase can replenish the lost telomeric DNA, a mechanism that plays a fundamental role in cancer growth, but there is no evidence that telomerase is a resilience mechanism for aging. Telomeres have been proposed to serve as a “molecular clock,” and short telomeres have been hypothesized to contribute to the aging process (Greider, 2010; Saretzki, 2018; Vera, Bernardes de Jesus, Foronda, Flores, & Blasco, 2012; Whittemore, Vera, Martinez‐Nevado, Sanpera, & Blasco, 2019). A 13‐year prospective study in the Baltimore Longitudinal Study of Aging reported that indeed, average telomere length shortens with aging, but the direction and magnitude of change are different in different circulating cells and extremely heterogeneous across individuals, with a substantial percentage of individuals showing average lengthening. Interestingly, significant amounts of telomere shortening were explained by decreased telomerase activity in the cells that express this enzyme, suggesting that measuring telomerase activity in human cells may be informative (Lin et al., 2015). Several reports indicate that short telomeres may be associated with central obesity (García‐Calzón et al., 2013; Mundstock et al., 2015), lifetime accumulation of stress (Epel et al., 2004; Osler, Bendix, Rask, & Rod, 2016; Puterman et al., 2016), increased risk of cardiovascular events (Baragetti et al., 2016; Hammadah et al., 2017), reduced immune response to influenza vaccination (Najarro et al., 2015), mortality (Batsis et al., 2017; Goglin et al., 2016; Heidinger et al., 2012), and several adverse health outcomes (Lin et al., 2015; Lorenzi et al., 2018; Lustig et al., 2017; Sanders & Newman, 2013). Genetic mutations associated with short telomeres have been shown to cause dyskeratosis congenita, pulmonary fibrosis, and several other severe medical conditions that are grouped under the definition of “telomere syndrome” (El‐Chemaly et al., 2018; Ungar et al., 2018). Different methods are available to measure telomere length in circulating cells, including restriction fragment analysis and fluorescence in situ hybridization. Observational studies using these techniques have reported contrasting results, and longitudinal studies have revealed erratic changes over time, possibly due to large measurement error (Berglund et al., 2016; Bischoff et al., 2006; Eerola et al., 2010; Lin et al., 2016; Müezzinler, Zaineddin, & Brenner, 2013; Solomon et al., 2014). Work is underway to establish an optimal “gold standard” assay for epidemiological studies (Behrens et al., 2017; Montpetit et al., 2014). At this stage, there is not enough evidence in the literature to consider measuring telomere shortening as a biological mechanism of aging or telomere length as a biomarker of biological aging. In general, the clinical relevance of measuring telomere length is unclear.

### 1.3 Cellular senescence

Cellular senescence is a stress response mechanism characterized by replication arrest and complex changes in morphology, chromatin organization, secretome, and expression of typical protein biomarkers (Muñoz‐Espín & Serrano, 2014; Rodier & Campisi, 2011). Conditions that trigger senescence include genomic instability, extreme telomere shortening, metabolic and proteostatic stress, reactive oxidative species (ROS), oncogene activation, mitochondrial dysfunction, epigenetic changes, and other mechanisms that have not been fully clarified (Childs, Durik, Baker, & van Deursen, 2015; Childs et al., 2017; López‐Otín et al., 2013). In general, these conditions trigger a response that activates the tumor suppressor genes p53, p16^Ink4a^, and p21 that utilize different pathways to induce cell cycle arrest (Hall et al., 2017; Liu et al., 2009). Most studies indicate that senescence‐induced replication arrest acts as a tumor suppression mechanism, but other physiological roles are emerging, including fetal organ development, wound healing, and aging (Baker & Petersen, 2018; Pratsinis, Mavrogonatou, & Kletsas, 2018; Wiley & Campisi, 2016; Zhang, Chen, Liu, Chen, & Liu, 2014). Irrespective of the nature of the senescence trigger, senescent cells develop a “senescence‐associated secretory phenotype” (SASP) and secrete pro‐inflammatory cytokines and chemokines, growth factors, and matrix proteases (Andriani et al., 2016; Coppé, Desprez, Krtolica, & Campisi, 2010; Strzyz, 2016). Notably, senescent cells become resistant to apoptosis and may persist in tissues for many years unless they are removed by the immune system, therefore interfering with tissue repair and regeneration (Kirkland & Tchkonia, 2017). It has been proposed that the accumulation of senescent cells and the negative effects of SASP proteins on intercellular matrix and on progenitor cells cause tissue degeneration and dysfunction, which may be a primary cause of aging and specific age‐related degenerative diseases, such as osteoarthritis, pulmonary fibrosis, atherosclerosis, diabetes, and Alzheimer's disease (Baker & Petersen, 2018; Bhat et al., 2012; Boccardi, Pelini, Ercolani, Ruggiero, & Mecocci, 2015; Diekman et al., 2018; Palmer et al., 2015; Waters et al., 2018). A recent study demonstrated that the number of cells expressing p16^Ink4a^ in biopsy specimens of intramuscular fat was independently correlated with lower muscle strength and worse walking performance (Justice et al., 2018). Although there is clear evidence that the burden of senescence increases with aging in human CD4^+^ lymphocytes, kidney epithelia, and skin, the quantification of senescence in vivo is complex because, in spite of the defined set of core features, heterogeneous forms of senescence develop according to different triggers and tissues (Koppelstaetter et al., 2008; Liu et al., 2009; Waaijer et al., 2012). Importantly, none of the characteristic biomarkers described above, including p53, p21, senescence‐associated β‐galactosidase, and SASP factors, are specific to senescence, and p16^Ink4a^ is not always present (Biran et al., 2017; Haferkamp et al., 2009; Laberge et al., 2012; Noren Hooten & Evans, 2017; Rodier & Campisi, 2011). Attempts to quantify senescent cell accumulation in humans from blood biomarkers assume that the SASP proteins dispersed in tissues spill over into circulation and may be detected there. Although none of these proteins are specific, jointly they could potentially identify a unique pattern that tracks the global burden of senescence across tissues or perhaps even show some specificity for their tissues of origin (Tanaka et al., 2018). The quantification of senescence in biopsies from different human tissues is an active area of research. Overall, quantification of senescence burden in humans is informative toward assessing biological aging, and measures based on cellular senescence are likely to enter soon into clinical research and practice.

### 1.4 Epigenetics

The term epigenetics encompasses the ensemble of mechanisms that modulate gene expression programs that adapt to environmental cues and define stable phenotypic characteristics from differentiated cell types (e.g., an adipocyte rather than a neuron). The three major epigenetic operators are DNA methylation, histone modification, and noncoding RNA. Among these three, a growing body of literature emphasizes the role of DNA methylation in aging and age‐related chronic diseases in humans (Gensous et al., 2017; Levine et al., 2018). In part, this is because DNA methylation is easily assessed in circulating cells and is relatively stable over time. In contrast, measuring histone posttranslational modification and noncoding RNA in humans is expensive, time‐consuming, not fully standardized, and amenable to rapid changes over relatively short time periods. In addition, while studies have related histone modifications and microRNA to cell senescence and diseases in animal models, whether these epigenetic mechanisms are drivers of biological aging in humans is uncertain (Bu, Wedel, Cavinato, & Jansen‐Dürr, 2017; Neault, Couteau, Bonneau, De Guire, & Mallette, 2017; Panda, Abdelmohsen, & Gorospe, 2017; Sidler, Kovalchuk, & Kovalchuk, 2017). Biochemically, DNA methylation is the addition of a methyl group to the 5th carbon of the pyrimidine ring of a cytosine (C) base juxtaposed to guanine (G) through a phosphate (p) bond (CpG), thus forming a 5‐methylcytosine (5mC). The presence of 5mC, especially at a promoter site, is generally believed to suppress gene transcription by blocking transcription factors from binding to promoter sequences, but accumulating evidence suggests that many other mechanisms are at play, including the control of transcriptional splicing (Avin, Umbricht, & Zeiger, 2016; Lev Maor, Yearim, & Ast, 2015; Young et al., 2005). DNA methylation is easily assessed in blood cells and tissues using microarrays, pyrosequencing, and whole‐genome bisulfite sequencing methods. As each CpG site can be differentially methylated in different cells, the site‐specific percent methylation of each CpG across the genome can be quantified. The percentage of 5hC at specific CpG sites can be used to derive an “epigenetic clock” that tracks closely with chronological aging (Hannum et al., 2013; Horvath, 2013). Their discovery has been confirmed by many studies across tissues, individuals, and populations, in addition to examining gestational age, and in longitudinal analyses (Horvath, 2013; Knight et al., 2016; Maierhofer et al., 2017; Quach et al., 2017; Sehl, Henry, Storniolo, Ganz, & Horvath, 2017). These findings demonstrate that some of the biological changes that occur with aging are not purely stochastic, but rather follow a predefined pattern that is constant across individuals and populations. Theoretically, the discrepancy between chronological and epigenetic clocks identifies individuals who are biologically older or younger than their chronological age. Consistent with this notion, “epigenetically older” individuals have a higher risk of developing several age‐related diseases and premature mortality for all causes and cardiovascular diseases (Chen et al., 2016; Marioni et al., 2015). In some studies, older epigenetic age has been associated with biomarkers of inflammation, as well as physical and cognitive function (Degerman et al., 2017; Gale et al., 2018; Levine et al., 2018; Ligthart et al., 2016; Marioni et al., 2015; Quach et al., 2017; Spiegel, Sewal, & Rapp, 2014). Unsurprisingly, the effect size for these associations is relatively small. As the CpG methylation sites included in epigenetic clock were selected based on chronological age, “discarded” CpG sites that deviate from chronological age are probably relevant in identifying accelerated or decelerated aging. In addition, most of the selection process of the relevant CpG sites has been cross‐sectional, which could be profoundly biased by secular trends. More recently, a second generation of epigenetic clocks was developed that uses a “phenotypic age” (PhenoAge) index for reference and/or is tuned to cardiovascular risk factors, including smoking (GrimAge), and is strongly predictive of mortality and a cadre of age‐related adverse health outcomes, including disability and dementia (Levine et al., 2018; Lu et al., 2019).

A recent literature suggests that hydroxymethylcytosine (5hmC), an oxidized form of 5‐methylcytosine (5mC) produced by Fe‐dependent dioxygenases named TETs (ten–eleven translocation) during demethylation, is a novel DNA epigenetic modulator with biological roles different from 5mC (Tahiliani et al., 2009). This view has been reinforced by the discovery of proteins showing a binding preference for 5hmC rather than 5mC (Mellen, Ayata, Dewell, Kriaucionis, & Heintz, 2012). Traditional bisulfite‐based assays for DNA methylation cannot distinguish 5mC from 5hmC, but new methods were recently developed for the regional detection and quantification of 5hmC (Szwagierczak, Bultman, Schmidt, Spada, & Leonhardt 2010; Terragni, Bitinaite, Zheng, & Pradhan, 2012). Differently from 5mC, abundance of 5hmC is highly variable across tissues, from less than 0.5% in the blood (Godderis et al., 2015) to close to 13% in the brain (Wen et al., 2014) where it is particularly high in mature neurons. Although the role of 5hmC has not definitively been established, contrary to 5mC that is thought to inhibit gene expression, 5hmC is enriched in coding regions of actively transcribed genes and some studies have shown positive correlations with expression levels (Branco, Ficz, & Reik, 2012; Colquitt, Allen, Barnea, & Lomvardas, 2013; Yu et al., 2012). There is evidence that hydroxymethylation increases with aging in several brain regions, including the hippocampus, while declining in peripheral mononuclear cells (Szulwach et al., 2011, Valentini et al., 2016). Brain hydroxymethylation has also been associated with age‐related neurodegenerative diseases such as Alzheimer's disease (Zhao et al., 2017). Whether information on hydroxymethylation and TET proteins in circulating cells or other tissues provides information on biological aging is unknown and is an active area of research.

**Figure 2:**
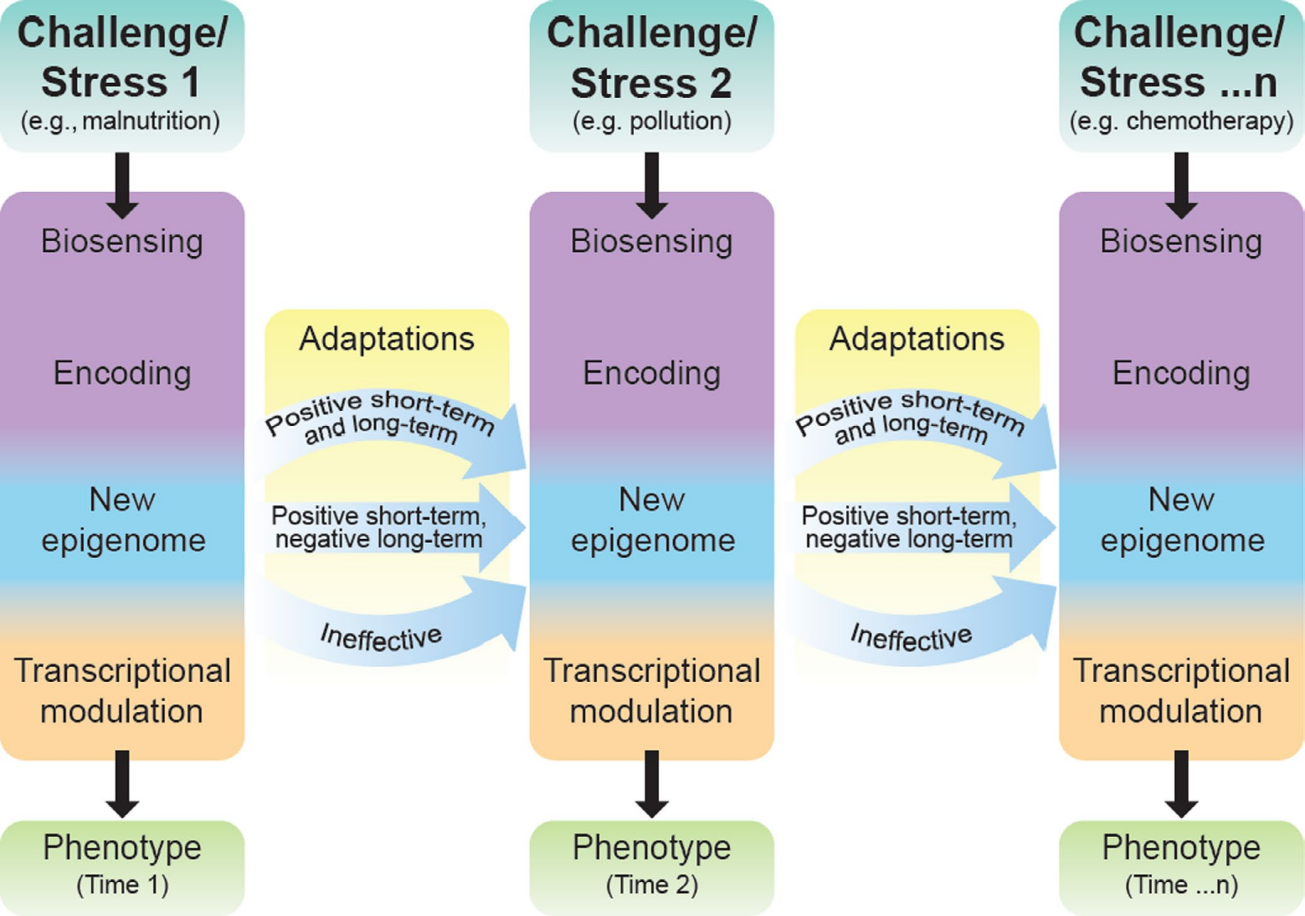
Epigenetic model of continuous transcriptional tuning leading to the aging phenotype. Long‐term adaptation within the lifespan requires epigenetic modulation of the transcriptional machinery. Environmental clues are read by specific biosensors and encoded into epigenetic changes that modulate transcriptional subroutines. The new epigenetic landscape is meant to be adaptive but may fail its purpose and become maladaptive in either the short or long term. Ineffective adaptation/compensation negatively impacts the rate of biological aging and, in turn, phenotypic and functional aging. In the scheme, we show only three cycles of epigenetic adaptation, at any point in time; the epigenetic landscape results from the sum of hundreds or even thousands of adaptive cycles that occur throughout life; and some more relevant than others. Importantly, very little is known about how environmental stresses are sensed and encoded into epigenetic changes

The development of epigenetic clocks is based on an agnostic statistical approach because biological mechanisms driving the clock are unknown. When these mechanisms are clarified, tools could be developed that would be even more useful for clinical applications. Also, the extent to which age‐related epigenetic changes can be considered evidence of damage or compensation remains unclear. Based on developmental theories, during the prenatal and early‐life periods, epigenetic mechanisms refine the genetic program to be optimally responsive to present and future environmental challenges. For example, massive epigenetic changes occur when food is scarce, and these changes may remain even when food becomes available later on, contributing to diabetes and metabolic syndrome (Bygren et al., 2014; Jiménez‐Chillarón et al., 2012; Jimenez‐Chillaron et al., 2006; Lorite Mingot, Gesteiro, Bastida, & Sánchez‐Muniz, 2017). The *theory of developmental origins of health and disease* hypothesizes that these early changes may be adaptive at the time they develop but may become maladaptive in later life, causing chronic diseases (Barker, Osmond, Winter, Margetts, & Simmonds, 1989; Ben‐Shlomo, Cooper, & Kuh, 2016; Pembrey, Saffery, & Bygren, 2014; Wadhwa, Buss, Entringer, & Swanson, 2009). The phasic approach to this theory can be extended to the continuum of the lifespan, and epigenetic changes may be considered as a cluster of predefined adaptive mechanisms that are implemented to counteract the effects of other typical biological changes that occur with aging. The essential elements of this theory are summarized in Figure 2. Research regarding the epigenetic clock clearly demonstrates that methylation in some specific CpG sites is reset at birth, as witnessed by the “zero” epigenetic age of cord blood (Knight et al., 2016). During aging, there is continuous epigenetic tuning of the predefined gene expression in response to environmental stress. This adaptive response, which likely occurs hundreds of times over the life course, may be fully adaptive or lead to negative consequences in subsequent years. Thus, in agreement with the *theory of developmental origins of health and disease*, over the life course physiological responses to stress are affected by all previous adaptations to stress already encountered, and the readout of this status is an epigenetic signature (Ben‐Shlomo et al., 2016). Thus, “epigenetic acceleration” would mark adaptive epigenetic changes that occur with aging earlier than average because of early imbalances between damaging and resiliency mechanisms. Interestingly, since methylation can be modified, interventions that “slow down” aging, thereby reducing the need for compensatory mechanisms, would also result in younger epigenetic age. Overall, DNA methylation is emerging as one of the most robust biomarkers of “biological aging” and represents a promising area for research that may be translated soon into clinical practice.

### 1.5 Mitochondrial function

Mitochondria are organelles found in all human cells, and their primary role is energy production through oxidative phosphorylation. They are also involved in signaling by producing ROS, as well as by regulating cellular metabolism, apoptosis‐programmed cell death, and other functions that are biologically important but cannot be reliably measured in vivo in humans (Gonzalez‐Freire et al., 2015). The mitochondrial theory of aging proposes that accumulation of damage to mitochondria and mitochondrial DNA (mtDNA) induces aging by reducing energy availability and increasing production of ROS that damage macromolecules (Harman, 1956, 1972, 2003). In humans, mitochondrial metabolic function is often studied in vitro in skeletal muscle by respirometry in permeabilized muscle fibers obtained through biopsies, as well as in vivo by phosphorous magnetic resonance spectroscopy (P^31^ MRS) (Lanza & Nair, 2010). Using both methods, it has been demonstrated that the degree of oxidative phosphorylation declines with aging in humans in the heart, skeletal muscle, and other tissues (Coen et al., 2012; Consolini, Ragone, Bonazzola, & Colareda, 2017; Fabbri et al., 2016; Gonzalez‐Freire et al., 2018; Holloway et al., 2018). Reduced mitochondrial function is associated with mobility decline in older persons, while the effect is mediated by a reduction of muscle strength (Zane et al., 2017). Currently, there are no measures of mitochondrial function in humans that are fully satisfactory. P^31^ MRS is noninvasive and reliable but is too expensive for large population studies, and this method only measures global skeletal muscle oxidative phosphorylation, which depends not only on the intrinsic mitochondrial function but also on the capacity of circulatory and microcirculatory system to deliver to mitochondria adequate amount of oxygen and oxidative substrates. Muscle biopsies are invasive but safe and allow for a variety of measurements—including direct mitochondrial respiration—as well as a wide range of biochemical assays that target different components of the energetic and biogenesis machinery, and the quantification of characterization of morphological changes using microscopy imaging techniques (Coggan, 1995; Hughes et al., 2015). Many of these indexes have been associated with aging and increased risk of chronic conditions (Consolini et al., 2017; Gonzalez‐Freire et al., 2018). Several assays are available for measuring ROS generation, antioxidant defense, and oxidative damage to macromolecules in blood, cells, and tissues (Dikalov & Harrison, 2014; Starkov, 2010). The significance of these oxidative stress biomarkers for aging is uncertain, as in many cases they have been studies in specific medical conditions and not in the context of aging studies (Al Shahrani, Heales, Hargreaves, & Orford, 2017; Hayashi & Cortopassi, 2015; Weber et al., 2017). Studies that use multiple mitochondrial biomarkers have revealed only a slight intercorrelation between the markers and aging, suggesting that they tap into different biological dimensions (Lara et al., 2015; Larsen et al., 2012; Marrocco, Altieri, & Peluso, 2017; Xia, Chen, McDermott, & Han, 2017). Recent data support the hypothesis that mtDNA copy number and degree of heteroplasmy—assessed in human blood cells and in tissue biopsies—provide information on mitochondrial physiology that is relevant for aging and age‐related diseases (McDermott et al., 2018; Moore et al., 2017; Zhang, Wang, Ye, Picard, & Gu, 2017). Both measurements can be utilized via PCR methods or, more recently, by derivation from genome sequencing data (Ding et al., 2015). High mtDNA copy number is considered to be a proxy measure of mitochondrial volume/function, and high mtDNA copy number in blood is associated with better health and survival among older persons, but the direction of this association may be reversed in certain conditions, such as diabetes (Mengel‐From et al., 2014; Moore et al., 2017). Humans have detectable levels of mtDNA‐acquired point mutations in circulating cells and whole blood and, notably, the burden of mutations increase with aging even when measured in inducible pluripotent stem cells (Kang et al., 2016; Qian et al., 2017). Many of these mutations are haploinsufficient or recessive and, when they reach a critical threshold of accumulation, can contribute to declining health in late life (Larsson, 2010; Wachsmuth, Hübner, Li, Madea, & Stoneking, 2016). Measures of mitochondrial physiology and function are powerful biomarkers of biological aging. However, they require extremely careful standardization. In particular, blood measurements may be affected by changes in circulating cells and high levels of mtDNA copy number can also indicate chronic tissue hypoxia (Eirin et al., 2016).

### 1.6 Proteostasis

The repair, recycling, and elimination of damaged macromolecules/organelles have emerged as key processes in maintaining cell integrity and function (Cuervo et al., 2005; Cuervo, Wong, & Martinez‐Vicente, 2010). These complex goals are accomplished through different mechanisms, such as chaperon‐dependent and chaperon‐independent autophagy, as well as protein biogenesis, folding, trafficking, and degradation (including proteasomal degradation; Kaushik & Cuervo, 2018; Morimoto & Cuervo, 2014; Wong & Cuervo, 2010). In animal models, autophagy and proteostasis become dysfunctional with aging. Rapamycin is an immunosuppressor that extends mammalian lifespans by inhibiting mTOR and stimulating autophagy. Genetic variants within core autophagy genes have been identified that contribute to human diseases, including hereditary spastic paraparesis, Parkinson's disease, and lysosomal storage disorders (Li et al., 2017; Settembre, Fraldi, Rubinsztein, & Ballabio, 2008; Wang et al., 2017). Beyond hereditary disease, evidence is emerging that autophagy becomes defective with aging and contributes to immunosenescence (Cuervo & Macian, 2014; Zhang, Puleston, & Simon, 2016). Accordingly, pretreatment with rapamycin analogs that inhibit TORC1 enhances immune function and reduces infections in the elderly (Mannick et al., 2014; Shavlakadze et al., 2018). Whether rapamycin or rapamycin analogs have potential for improving healthspan and lifespan in humans is unclear, and their potential side effects are of significant concern. Rapamycin analogs that selectively target TORC1, which should have less side effects, have been proposed for treatment of diseases of aging (Arriola Apelo & Lamming, 2016; Bjedov et al., 2010; Chi et al., 2015; El‐Chemaly et al., 2017; Miller et al., 2010).

Other compounds that modulate autophagy have shown anti‐aging properties, including the polyamine spermidine, the natural polyphenol resveratrol, and the gut bacterial product urolithin A. Tissue levels of spermidine decline with age in model organisms and in humans, although they are unusually high in healthy nona/centenarians (Eisenberg et al., 2009; Gupta et al., 2013; Pucciarelli et al., 2012). Spermidine administration increases lifespan and healthspan of multicellular model organisms, at least in part though TORC1 inhibition and enhancement of autophagy. Indeed, blockage of autophagy removes most positive effects of spermidine (Madeo, Eisenberg, Pietrocola, & Kroemer, 2018). Several lines of research suggest that resveratrol enhances autophagy and, through this mechanism, protects against multiple age‐related chronic diseases and increases longevity in mice on a high‐fat diet (Agarwal & Baur, 2011). Mechanisms by which resveratrol induces autophagy are still not fully elucidated but certainly involve both mTOR inhibition and histone deacetylation through the AMPK/SIRT1 signaling pathway (Lee et al., 2008). Interestingly, the combination of spermidine and resveratrol shows synergistic effects on autophagy induction (Morselli et al., 2011).

Urolithin A is a metabolite produced by gut microbiota from compounds found in many fruits and vegetables. Urolithin A has been shown to induce mitophagy in cell cultures, increase longevity in nematodes, and prevent age‐related muscle impairment in mouse models (Ryu et al., 2016). Administration of urolithin A in healthy, sedentary elderly individuals is followed by changes in muscle mitochondrial gene expression that are suggestive of improved mitochondrial and cellular health (Andreux et al., 2019).

Developing assays for autophagy in humans is challenging. Static measures of autophagosome accumulation based on quantification of LC3, an antigen that is only present in autophagosomes, are relatively simple, yet notoriously unreliable. In contrast, measures that track the dynamic flux of the autophagic process, by quantifying accumulation of autophagosome cargo upon inhibition of lysosomal proteolysis, are more reliable and the only suitable assay to discriminate whether increase abundance of autophagosomes is due to increase biogenesis or to reduced clearance (Klionsky, 2014; Yoshii & Mizushima, 2017). Recent studies suggest that adequate quantification of autophagy requires multiple approaches, most of which are expensive, labor‐intensive, and low‐throughput. Thus far, only a few studies provide evidence that autophagy becomes dysfunctional with aging, and a recent study shows that autophagy appears to be better maintained in members of families with extended longevity and positively correlates with improved T‐cell function (Raz et al., 2017). Similarly, no high‐throughput method is available for assessing proteostasis. Recently, a new measure has been developed that uses tetraphenylethene, a fluorescent dye, to label the free cysteine thiols that are normally hidden in the core of properly folded globular proteins and are uncovered by misfolding (Chen et al., 2017). Also, using prolonged starvation in human volunteers, Pietrocola et al. developed a method to assess autophagy in circulating leukocytes. They could detect enhanced autophagic flux in human neutrophils cultured in the presence or absence of leupeptin (Pietrocola et al., 2017). Although these methods are promising, further development and validation in human cells is needed before these assays can be used in clinical studies. Overall, mechanisms that handle repair, recycling, and eliminating damaged macromolecules/organelles could act as strong biomarkers of biological aging and would be extremely useful in clinical application, but better assessment methods need to be developed.

### 1.7 Stem cell exhaustion, deregulated nutrient sensing, and altered intercellular communication

These three “hallmarks of aging” have been combined in this section because their impact on age‐related diseases, healthspan, and longevity in humans has not been sufficiently characterized. Stem cell exhaustion has been postulated to play a primary role in aging as it interferes with self‐renewal of differentiated cells in tissues and organs, slowly curtailing function (Ren, Ocampo, Liu, & Izpisua Belmonte, 2017). Small cross‐sectional studies have provided some evidence that hematopoietic stem cells in humans accumulate DNA damage, possibly leading to reduced proliferative potential (Beerman, 2017; de Haan & Lazare, 2017). Studying the effect of aging on stem cells in humans is difficult. Hematopoietic stem cells, satellite cells, and epidermal stem cells represent the only easily accessible material, but their isolation is still problematic and only yields small quantities (Ahmadbeigi et al., 2013; Hinken & Billin, 2018; Lavker & Sun, 2000; Liu, Cheung, Charville, & Rando, 2015; Moestrup, Andersen, & Jensen, 2017; Rossi, Challen, Sirin, Lin, & Goodell, 2011). Overall, despite the great enthusiasm for using stem cells to treat many age‐related disease, data on changes with human aging in stem cell numbers, characteristics, and replication potential are still limited (Dexheimer, Mueller, Braatz, & Richter, 2011; Eichler et al., 2017; Fan et al., 2010; Golpanian et al., 2017, 2016; Hare et al., 2012; Jim et al., 2016; Li, Chen, Han, & Fu, 2010; Pang et al., 2011; Rigotti et al., 2016; Tompkins et al., 2017; Volarevic et al., 2011, 2018; Zhang et al., 2011). Understanding whether changes in stem cells biology are important for aging remains an important and promising question, and research in this field is warranted.

Nutrient sensing in humans is important for aging and longevity based on the extraordinary effectiveness of caloric restriction in increasing longevity and healthspan in animal models, including mammals (Anderson, Le Couteur, & de Cabo, 2017). Whether this concept can be transformed into empirical measures in humans remains to be elucidated. Similarly, the concept of “intercellular communication” is so generic as to encompass almost any known physiological mechanism. This concept will be revisited when discussing “inflammation,” which may be a special case of “intercellular communication” that is dysregulated with aging and predicts several adverse health outcomes in humans, as well as multimorbidity (Bektas, Schurman, Sen, & Ferrucci, 2018; Fabbri et al., 2014; Friedman, Christ, & Mroczek, 2015; Sanada et al., 2018).

## 2 CONNECTING THE BIOLOGY OF AGING WITH AGE‐ASSOCIATED MULTIMORBIDITY

Based on information in the section above, developing a proxy measure of biological aging for humans still requires work but is a very dynamic and promising area of investigation with strong potential for translation. Some of the measures described—namely mitochondrial function, DNA methylation, and, to a lesser extent, cellular senescence and autophagy—are ready to be implemented based on several epidemiological studies, although refinements are always possible (Capri et al., 2015; Choi et al., 2016; Cohen, Morissette‐Thomas, Ferrucci, & Fried, 2016; Jylhävä, Pedersen, & Hägg, 2017; Jylhävä et al., 2014; Kananen et al., 2016; Kent & Fitzgerald, 2016; Kim & Jazwinski, 2015; Levine et al., 2018; Li et al., 2018; Marioni et al., 2019; Marttila et al., 2015; Putin et al., 2017; Sillanpää et al., 2018). Measures of telomere length are hampered by noise and wide longitudinal variations that cannot be explained by health events and at this stage are not useful for measuring biological age (Arai et al., 2015; Jodczyk, Fergusson, Horwood, Pearson, & Kennedy, 2014; Tomaska & Nosek, 2009). New methods are being developed, some of which are focused on detecting the DNA damage response (a typical marker of critical telomere shortening) may yield better results (Choi, Kim, Kim, Kemp, & Sancar, 2015; Hewitt et al., 2012; Rossiello et al., 2017). Senescence has been studied successfully in T lymphocytes, skin, and intramuscular fat, and high‐throughput methods will be available soon (Evangelou et al., 2016; Lozano‐Torres et al., 2017). In addition, specific patterns of circulating proteins may exist that indirectly estimate the burden of senescence (Angelini et al., 2017; Hoffman, Lyu, Pletcher, & Promislow, 2017; Kadota et al., 2018; Menni et al., 2014; Tanaka et al., 2018; Yousefzadeh et al., 2017). Similarly, measures of autophagy are routinely used in mammalian studies and should be applicable to humans (Klionsky, 2014; Klionsky, Cuervo, & Seglen, 2007; Menzies, Moreau, Puri, Renna, & Rubinsztein, 2012). For the other hallmarks, the development of a reliable and valid test is less advanced and will take time.

**Figure 3:**
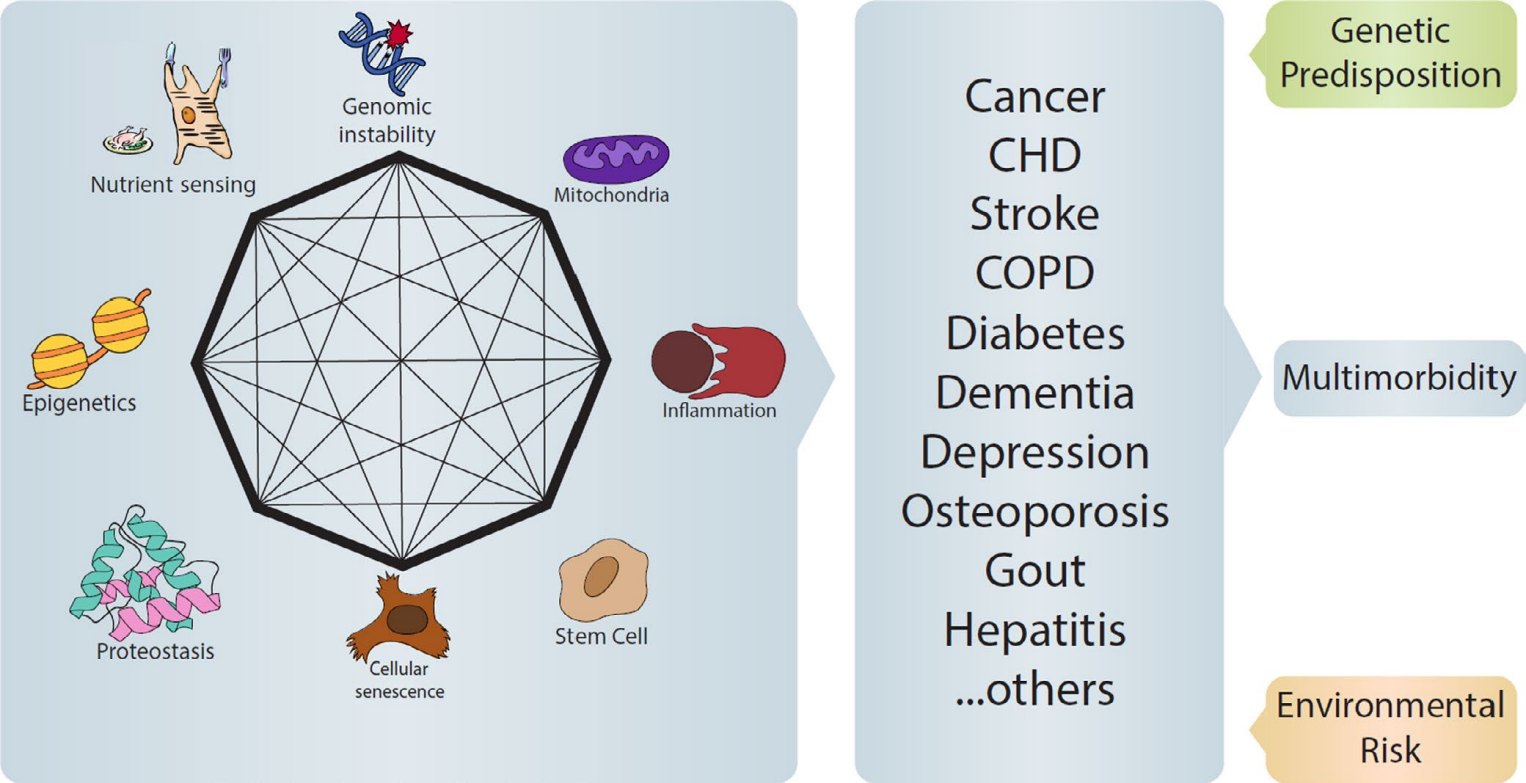
The hallmarks of aging are specific biological mechanisms that drive the rate of biological aging. Emerging research reveals that these different mechanisms are strongly interconnected and, therefore, impairment in one mechanism involves the others. In the figure, the octagon and lines within represent evidence for connections between the different mechanisms. The evidence reported is not exhaustive of the literature connecting the hallmarks. According to the *geroscience* hypothesis, failure in this network of homeostatic mechanisms affects the pace of aging and, in turn, causes a growing susceptibility to diseases. The specific combination of coexisting diseases that occur in each individual depends on their genetic background, as well as exposure to environmental and behavioral risk factors. The resulting multimorbidity is a major cause of disability. Notably, if the number of coexisting diseases is a major proxy biomarker of the pace of aging, it is unsurprising that the number of diseases rather than specific combination is the strongest risk factor for disability

Multiple lines of evidence suggest that the measures listed above are associated with the severity of multimorbidity but, except for the epigenetic clock, this association has not yet been clearly established. Logically, none of the measures described above represent an exhaustive measure of biological aging and, therefore, new aggregate measures are needed that leverage differences and complementarities of the various biomarkers. To accomplish these goals, the hallmarks of aging should be assessed in a group of individuals that is reasonably sized and enough dispersed across the lifespan to represent the variability of biological age in the general population. Initially, it will be important to evaluate the intercorrelation between these measures, as there is currently evidence that the hallmarks of aging are interconnected (Figure 3). Each numbered arrow in the left portion of Figure 3 refers to a piece of evidence that failure of a certain mechanism leads to impairment in others, a notion that is strengthened by emerging evidence in recent literature, although most is derived from animal studies (Acosta et al., 2013; Chang et al., 2015; Childs, Li, & van Deursen, 2018; García‐Prat et al., 2016; Gonzales‐Ebsen, Gregersen, & Olsen, 2017; Hall et al., 2017; Herranz & Gil, 2018; Kang et al., 2015; Ligthart et al., 2016; Mills, Kelly, & O'Neill, 2017; Moreno‐Blas, Gorostieta‐Salas, & Castro‐Obregón, 2018; Netea‐Maier, Plantinga, Veerdonk, Smit, & Netea, 2015; Wiley et al., 2016). Clarity is needed in determining if the hallmarks of aging are multifaceted expressions of the same core process or if they evolved independently, as interventions would either have to target each single mechanism or could address one mechanism with synergistic benefits on the others. A simple cross‐sectional correlation may not be optimal, as different manifestations of biological aging may occur according to different time schedules, some mechanism preceding others (Ferrucci, Levine, Kuo, & Simonsick, 2018). Thus, these measures needed to be examined using exploratory “lagged analysis” in a longitudinal perspective. Interestingly, all of the “hallmarks of aging” cited above directly or indirectly cause an inflammatory state, suggesting that the pro‐inflammatory state observed in many older persons may reflect the burden of biological aging (Ferrucci et al., 2005; Franceschi & Campisi, 2014; Fulop et al., 2018). Consistent with this hypothesis, inflammation measured by circulating levels of IL‐6 is the only known cross‐sectional and longitudinal predictor of multimorbidity and one of the strongest predictors of incident mobility loss and disability in activities of daily living (Fabbri et al., 2014; Ferrucci et al., 1999, 2002). Mobility loss, disability, and mortality could be used as reference outcomes to calibrate an index of biological aging as a weighted aggregated, predictive measure. However, while the “functional” outcomes are critical for quality of life in the elderly, they occur late in life and fail to capture the initial changes of biological aging at younger ages. Focusing on multimorbidity is a very promising approach, especially as the pace of biological aging and the development of subclinical pathologies are the primary forces behind increased susceptibility to disease (Fabbri et al., 2014). The rate of aging translates into different patterns of multimorbidity due to specific combinations of genetic susceptibility and environmental stress (Figure 3). Finally, as aging is a dynamic construct, the strength of any index of biological aging should be validated longitudinally by demonstrating that the accelerated progression of “biological aging” is paralleled by an accelerated deterioration in the phenotypic and functional dimensions of aging.

## 3 THE TRANSLATIONAL VALUE OF ASSESSING BIOLOGICAL AGING

Substantial investment is necessary to develop an estimator of biological aging that is robust, precise, reliable, and sensitive to change. Thus, a fair question is whether such a titanic project is worth the effort and cost. The answer is YES, without hesitation. Developing an index of biological aging is perhaps the most critical milestone required to advance the field of aging research and, especially, to bring relieve from the burden of multimorbidity and disability in an expanding aging population. Ideally, these measures would be obtained by running tests using blood samples without performing a biopsy, preferably quickly and at low cost. An index of biological aging could be used to empirically address the geroscience hypothesis: “Is biological aging is the cause of the global susceptibility to disease with aging.” Data collected longitudinally—ideally in a life course epidemiological study—could then be used to test if individuals that accumulate coexisting diseases faster than in the general population also have accelerated biological aging. Similarly, these data could be used to test if individuals who are biologically “older,” independent of chronological age, are at a higher risk of developing different medical or functional conditions that do not share physiological mechanisms. Once validated, the fundamental basis of biological aging can be used to probe deeper into questions related to the mechanisms of aging, such as the following: Are there genetic traits that are associated with faster or slower biological aging? Are there “hallmarks” that are better at capturing biological aging at different stages of life?

These questions have immense relevance for geriatric medicine. Despite the rising emphasis on prevention, most current medical care is dedicated to diagnosing and managing diseases that are already symptomatic, which does not address the underlying issues related to geriatric health conditions. By understanding the intrinsic mechanisms of biological aging, including damage and resilience, medical professional will be able to best orient and prescribe therapeutic choices. These mechanisms are summarized in Table 1 according to the current state of knowledge. The first column lists measures of damage for each one of the hallmarks of aging, the second lists the compensatory measures that we would like to have available, and the third lists the compensatory measures that are currently available. Clearly, the current ability to measure biological compensations and resilience is very limited, although most are vital to human health. In fact, it has been proposed that chronic diseases, especially those that emerge in old age, may be cross‐classified based on their dependence on the force of the “noxa patogena” and the robustness of resilience.

**Table 1:** Biomarkers of “damage” and “compensation” for the different hallmarks of aging

Hallmark	Damage	Resilience (compensation) response	Measures
Genomic instability	Somatic mutations (including in stem cells) Inappropriate clonal expansion DNA modifications (8‐oxoG, gammaH2AX, etc.)	DNA repair mechanisms Cellular checkpoint responses (e.g., cell cycle arrest, senescence, apoptosis) Integrity of replication fidelity mechanisms Antioxidant mechanisms	Single‐cell/clonal NGS Tests of DNA repair mechanisms Measures of DNA modifications
Telomere shortening	Telomere dysfunction in mitotic cells, stem cells, and germline cells	Telomerase Cellular checkpoint responses	Telomere length Markers of DNA damage response Telomerase activity
Cellular senescence	Arrested cell proliferation SASP, chronic inflammation	Immune clearance of senescent cells SASP suppression by mTOR signaling Prevention of irreversible senescence	Senescent markers in blood and tissue SASP proteins in blood and tissue
Epigenetic changes	Inappropriate increase or decrease in DNA methylation at specific sites Inappropriate increase or decrease in specific histone modifications Maladaptive epigenetic changes	Epigenetic maintenance system Mechanism of epigenomic reprogramming Adaptive changes in epigenetic markers Suppression of negative and enhancement of positive transcriptional programs	Methylation Histone acetylation
Mitochondrial dysfunction	Impaired respiration/ox/phosph Ineffective mitochondrial biogenesis Ineffective mitochondrial recycling Mitochondrial disorganization ROS‐mediated oxidative damage	Mitochondrial biogenesis Mitochondrial remodeling (fission/fusion cycles), mitophagy Maintained mtDNA replication fidelity Antioxidant defenses	Mitochondrial volume/number/shape Mito respiration P^31^ MRI spectroscopy Markers of biogenesis mtDNA copy number and haplotypes
Decreased autophagy, proteostasis	Increased damaged/misfolded proteins Decreased protein function Permanence of unrecycled proteins/organelles Cell death due to increased autophagy	Activity of macro‐, micro‐, and chaperone‐mediated autophagy‐related proteins Enhanced signaling pathways (e.g., mTOR signaling) that regulate levels of autophagy	Autophagy markers and flux (+ TEM) Chaperon proteins
Stem cell exhaustion	Reduced stem cell number Decreased proliferative capacity Decreased differentiation capacity	Reprogramming? Quiescence maintenance	Proliferative capacity in vitro Resistance to stress

Note

The second column lists measures of damage, some of which are already feasible in humans, while others are only theoretically feasible. The third column lists measures of resilience that would be theoretically desirable, while the fourth column lists measures that are currently feasible. Importantly, regarding many of the available measures, understanding if they reflect damage or compensation requires further investigation.

Abbreviations: NGS, new‐generation sequencing; SASP, senescence‐associated secretory phenotype; TEM, transmission electron microscopy.

The approach described above is not too farfetched from our experience. Hopefully, we all take good care of our cars before they break or malfunction; we make sure that the water an oil levels are ok, that the brake pads are not consumed, that the pressure in the tires is according to factory recommendations. We carefully follow maintenance schedule because we want to maximize the healthy life of our cars and avoid expensive repairs and replacements. Shouldn't we pay the same attention to our bodies? In the field of geriatrics, the situation is even more extreme and often patients come to the clinic when they are already affected by multiple diseases, have lost their autonomy, and have economic and social constrains. In other words, they come to observation when all the mechanisms of compensation and resilience are exhausted. Despite these odds, geriatricians sometime make miracles, but certainly not often enough. The possibility of measuring biological aging swaps this perspective and allows the assessment of health status at a time when our physiology is still resilient, there are still no symptoms, and interventions are more likely to be effective.

A robust biomarker of biological aging would have benefits beyond the early identification of persons who age “faster” than others. First, the genetic, environmental, and behavioral risk factors associated with accelerated aging could be identified. Then, longitudinal studies could be utilized to identify specific time points at which the trajectories of aging change and relate to those other health‐related triggers, such as the exposure to pollution associated with moving to a different city. As biological aging is the primary cause of resilience loss, measuring damage and compensation may help in determining between interventions with potentially serious side effects. Longitudinally, a marker of aging could be used to track if interventions with similar efficacy toward a specific target affect the “speed of aging” differently, which may impact accelerated declines in health. This approach could be used to both refine choices in alternative therapies and develop new medications in order to avoid damage accumulation or curtail compensatory mechanisms. Clinical trials then can be designed to specifically target the speed of aging, the underlying causes of multimorbidity, or both as the primary outcomes of interest. The list of interventions is almost limitless, even without considering the many other applications that are currently unknown and will only become evident as the field progresses.

## 4 MULTIMORBIDITY AND THE ART OF GERIATRICIANS

A primary focus in geriatric medical is the management of patients affected by multiple coexisting, chronic diseases, as well as physical and cognitive impairments. Indeed, geriatric patients typically have a long list of diagnoses, prescriptions, impairments, social problems, and financial constraints, often presenting a medical dilemma with no clear solution. Most clinical guidelines focus on one disease and, in only exceptional and recent cases, on diseases that belong to the same organ system (Jani et al., 2017; Moreno, Mangione, Kimbro, & Vaisberg, 2013; Spaak, 2017). This is in spite of the fact that co‐occurrence of two or more chronic diseases is the most prevalent medical condition in persons 65 or older (Cesari, Pérez‐Zepeda, & Marzetti, 2017; Fabbri et al., 2015; Guiding Principles for the Care of Older Adults with Multimorbidity: An Approach for Clinicians, 2012; Tisminetzky et al., 2017; Vetrano et al., 2017). Daily, geriatricians are faced with overwhelming complexity, requiring powerful tools: an exhaustive knowledge of medicine and physiology, the ability to evaluate from a list of diseases to choose from possible therapies, and a strong focus on quality of life and on patient preferences. Unfortunately, they are limited with little undersatnding of the biological basis for aging. If multimorbidity is a stochastic assemblage of separate pathologies, the resulting number of syndromes exceeds any serious attempt at classification, which is an essential prerequisite for tailored interventions. Thus, caring for older patients becomes a cyclic process, involving a sequence of trials and errors that are driven by a mixture of knowledge, experience, and intuition.

## 5 MULTIMORBIDITY AS AN EXPRESSION OF BIOLOGICAL AGING

The emerging field of *geroscience* presents a hopeful approach to multimorbidity, which aims to understand the relationship between biological aging and age‐related diseases at the molecular level. The traditional approach to studying of aging is rooted in a clear‐cut distinction between aging and diseases, while the geroscience paradigm intimately connects the molecular mechanisms of aging with the rising susceptibility to diseases. This may explain why the number of coexistent chronic diseases tends to increase geometrically with aging in both men and women (Fabbri et al., 2015; GBD, 2016 Disease, & Injury Incidence & Prevalence Collaborators, 2016; Guiding Principles for the Care of Older Adults with Multimorbidity: An Approach for Clinicians, 2012; He et al., 2018; Melis, Marengoni, Angleman, & Fratiglioni, 2014; Niccoli & Partridge, 2012; Rae et al., 2010; Rocca et al., 2014; St Sauver et al., 2015). This conceptual shift on the origin of age‐related multimorbidity opens new, previously unexplored opportunities for research and clinical care in older persons. Importantly, if the core mechanisms of aging can be identified, they could be targeted for interventions aimed at preventing multimorbidity and disability, while also improving the quality of life in old age.

To explain the development of this new science, the conceptual paradigm of geroscience needs to be fully explored. Time is the most “robust” and “precise” metric of aging; however, the chronological dimension presents intrinsic problems due to the magnitude of anatomical and physiological changes that occur with aging in a single time unit (e.g., one year), which can be quite heterogeneous.

## 6 CONCLUDING REMARKS

Progress in research is not linear. Periods characterized by rates of incremental knowledge are interlaced with “eureka” moments as milestone discoveries suddenly open new possibilities that thrust research and knowledge to a higher level. Galileo's use of the telescope to explore the stars, Kary Mullis's description of polymerase chain reaction, and Edwin Hubble's demonstration that the universe is expanding are just few examples of these moments. The field of aging research is living one of those magical moments. Finding a reference metric for the rate of biological aging is key to understanding the molecular nature of the aging process. Defining and validating this metric in humans opens the door to a new kind of medicine that will overcome the limitation of current disease definitions, approaching health in a global perspective and bringing life course preventative measures to the center of attention.

## CONFLICT OF INTEREST

None.
